# Lung and Colon Cancer Detection Using a Deep AI Model †

**DOI:** 10.3390/cancers16223879

**Published:** 2024-11-20

**Authors:** Nazmul Shahadat, Ritika Lama, Anna Nguyen

**Affiliations:** Department of Computer and Data Sciences, Truman State University, Kirksville, MO 63501, USA; ritikatheeng2002@gmail.com (R.L.); nguyenthucanh.35@gmail.com (A.N.)

**Keywords:** 1D CNN, squeeze-and-excitation networks, RCN, lightweight model, lung and colon cancer detection, lung cancer detection, colon cancer detection, cancer detection, histopathological images, image classification, deep learning

## Abstract

Lung and colon cancers are among the leading causes of cancer-related mortality worldwide. Early and accurate detection of these cancers is crucial for effective treatment and improved patient outcomes. False or incorrect detection is harmful. Accurately detecting cancer in a patient’s tissue is crucial to their effective treatment. While analyzing tissue samples is complicated and time-consuming, deep learning techniques have made it possible to complete this process more efficiently and accurately. As a result, researchers can study more patients in a shorter amount of time and at a lower cost. Much research has been conducted to investigate deep learning models that require great computational ability and resources. However, none of these have had a 100% accurate detection rate for these life-threatening malignancies. Misclassified or falsely detecting cancer can have very harmful consequences. This research proposes a new lightweight, parameter-efficient, and mobile-embedded deep learning model based on a 1D convolutional neural network with squeeze-and-excitation layers for efficient lung and colon cancer detection. This proposed model diagnoses and classifies lung squamous cell carcinomas and adenocarcinoma of the lung and colon from digital pathology images. Extensive experiment demonstrates that our proposed model achieves 100% accuracy for detecting lung, colon, and lung and colon cancers from the histopathological (LC25000) lung and colon datasets, which is considered the best accuracy for around 0.35 million trainable parameters and around 6.4 million flops. Compared with the existing results, our proposed architecture shows state-of-the-art performance in lung, colon, and lung and colon cancer detection.

## Simple Summary

Lung and colon cancer are among the leading causes of cancer-related deaths globally, emphasizing the critical need for early and precise detection to improve treatment and patient outcomes. This research introduces a novel deep learning model for efficient lung and colon cancer detection, aiming to address the limitations of existing computationally intensive models. This study proposes a lightweight, parameter-efficient model suitable for mobile devices, utilizing a 1D convolutional neural network enhanced with Squeeze-and-Excitation layers. By achieving 100% accuracy on a large dataset of histopathological images, this research provides a significant advancement in cancer detection technology. The findings hold the potential to revolutionize medical diagnostics by enabling faster, more accessible, and reliable cancer screening, potentially leading to earlier interventions and improved survival rates.

## 1. Introduction

Cancer is a disease in which cells grow uncontrollably and spread throughout the body [1]. Trillions of cells are living in a healthy body. Normal cells unceasingly reproduce only if needed and instructed by other cells, ensuring fixed sizes of each tissue. On the other hand, cancer cells, with their ability to migrate and invade nearby tissues, increase the masses of tissue [2]. These cells can develop tumors that can be malignant or benign. Cancerous tumors, which are also known as malignant tumors, invade neighboring tissues and travel throughout the body to generate new tumors (a process known as metastasis). Many malignancies produce solid tumors, while blood cancers, such as leukemia, do not. Benign tumors do not spread to or infect surrounding tissues. They barely reproduce after removal, although malignant tumors do. However, benign tumors can grow quite large. Some, like benign brain tumors, can cause severe symptoms or even death [3].

According to the World Health Organization (WHO), there were estimated to be about 20 million new cancer diagnoses and 9.7 million fatalities in 2022 [4]. After a cancer diagnosis, an estimated 53.5 million people are expected to survive for five years. About one in every five individuals will experience cancer in their lifetime, and the disease is fatal for one in every nine men and one in every twelve women. According to an estimation from the IARC’s Global Cancer Observatory, the three most common cancer types worldwide in 2022 were lung, breast, and colorectal cancers. The data covered 185 nations and 36 different types of cancer, and showed that ten specific cancers account for around two-thirds of all new cases and fatalities worldwide. Lung cancer led the list, accounting for 2.5 million new cases, or 12.4% of all new cancer cases. Breast cancer was second with 2.3 million cases (11.6%), followed by colorectal cancer with 1.9 million (9.6%). Other primary cancers were prostate cancer (1.5 million cases) and stomach cancer (970,000 instances). In terms of mortality, lung cancer was the leading cause, accounting for 1.8 million fatalities (18.7% of total cancer deaths), followed by colorectal cancer, liver cancer, breast cancer, and stomach cancer. The high incidence of lung cancer, particularly in Asia, is associated with continued tobacco use. There are significant disparities in cancer incidence and death between sexes. Breast cancer was the most often diagnosed cancer and the leading cause of cancer mortality in women, whereas lung cancer held both distinctions in males. Prostate and colorectal cancers are the most common diagnoses in men after lung cancer, and liver and colorectal cancers are the second and third leading causes of death, respectively. In women, lung and colorectal cancers are the second and third most common causes of new cases and fatalities, respectively. In 2024, it is expected that there will be 2,001,140 new cancer cases in the United States, with 611,720 deaths from the disease. For men, prostate, lung, and colorectal cancers are predicted to account for 48% of all cancer cases. Similarly, breast, lung, and colorectal cancers are expected to account for 51% of all diagnoses in women, indicating their widespread influence on the population [3].

Cancer cells differ significantly from normal cells. They can develop without external growth signals, whereas normal cells require such signals to divide. They also disregard signals that generally stop cell division, trigger apoptosis, or programmed cell death. Furthermore, cancer cells invade neighboring tissues and can spread to other areas of the body, but normal cells stick to their specific territory and rarely move. They can stimulate the growth of blood vessels, leading to tumors and providing a continuous supply of nutrients and oxygen while assisting in waste removal. These cells can also evade the immune system, which typically destroys aberrant cells, and influence immunological responses to promote their survival and growth. Furthermore, cancer cells often have significant chromosomal changes, including duplication and deletion, and may have double the number of chromosomes as normal cells. They also absorb and utilize nutrients differently, enabling faster growth and multiplication compared to other cells [3].

Carcinomas, the most prevalent type of cancer, are caused by epithelial cells that cover both the internal and external surfaces of the body. Under a microscope, these cells generally appear to be column-shaped. Various carcinomas are called after the kind of epithelial cell involved. Adenocarcinoma develops from epithelial cells that produce fluids or mucus and is common in breast, colon, and prostate cancers. Basal cell carcinoma begins in the basal layer of the epidermis, the skin’s outermost layer. Meanwhile, squamous cell carcinoma develops from squamous cells, which are flat and scale-like and found just beneath the skin’s surface and lining various organs such as the stomach, intestines, lungs, bladder, and kidneys. This form is also known as epidermoid carcinoma. Adenocarcinoma, squamous cell carcinoma, and large cell carcinoma are identified as non-small cell lung cancer (NSCLC) due to their similarities in treatment and prognoses. They accounted for 85% of lung cancer types. On the other hand, small cell lung cancer (SLCL) accounted for up to 15%. SCLC grows and spreads much faster than NSCLC, and when patients are diagnosed, the cancer will have already spread beyond the lungs [5].

Colorectal cancer occurs when cells in the colon or rectum grow out of control, which is also known as “colon cancer”. Abnormal growths, known as polyps, can arise in the colon or rectum. Over time, certain polyps may develop into cancer. Cancer cells spread from the innermost layer of the colon and rectum’s wall to the outer layers. Even though colorectal cancer can be completely treated if detected early, it can still spread to other organs, especially the lungs, which is known as lung metastasis. The American College of Surgeons found that of 50% colon cancer patients, in 18% of them cancer had spread to the lungs. That is, a patient with colon cancer might have a high chance of having lung cancer synchronously [6].

Symptoms can manifest in the very early stages of cancer. However, they are often not significantly noticeable as these symptoms are commonly mistaken for a common cold or flu, displaying signs such as loss of appetite and coughing. This underscores the importance of regular screening tests to detect and remove abnormalities such as polyps before they develop into cancer. Imaging tests or histopathology images, such as X-rays, ultrasound, MRI, and CT scans, create detailed internal body images. These tests serve multiple purposes: identifying potential cancer locations, measuring cancer spread, assessing ongoing treatment effectiveness, and monitoring for cancer reappearance post-treatment. A computed tomography (CT or CAT) scan, which uses X-rays to produce accurate cross-sectional pictures, is particularly useful in diagnosing whether colon cancer has spread to lymph nodes or essential organs such as the liver, lungs, or others. In the past, doctors had to go through a lengthy and laborious procedure to review histological pictures and identify cancer cases; however, with the continuous development of technology, this process may now be completed much faster with the vital assistance of artificial intelligence (AI) [7].

AI has shown exceptional abilities in medical diagnosis, analyzing various tests such as CT scans, MRI scans, X-rays, blood tests, and biopsies using AI techniques. However, this paper also analyzes test images using our proposed architecture. The diagnostic process involves collecting samples and integrating and interpreting information to provide a diagnosis, which forms the basis for implementing the appropriate treatment plan. Given that people are prone to errors, it is not surprising that overdiagnosis is more common among patients, leading to unnecessary treatment and impacting health and the economy [8]. AI can significantly aid the healthcare system in timely and accurately identifying and diagnosing diseases. A branch of AI, machine learning (ML), focuses on using data as input resources [9] and performs tasks without explicit programming. Healthcare experts implement the most recent machine learning in triage to highlight abnormal cells and prioritize life-threatening patients [10]. Applying specified mathematical functions produces a result (classification or regression) often impossible for people to achieve [8]. The evolution of deep learning (DL) algorithms has enabled machines to evaluate complicated, high-dimensional data, such as images, multidimensional anatomy scans, and videos. DL, a subset of machine learning (ML), is a collection of algorithms meant to replicate the structure and function of the human brain. This improves their capacity to comprehend and learn from massive quantities of data [11]. DL algorithms can identify patterns and abnormalities that may not be visible to the human eye. In recent decades, DL has optimized using artificial neural networks (ANNs), support vector machines (SVMs), etc., to improve its pattern identification abilities.This paper introduces a novel mobile-embedded deep learning architecture with a 1D convolutional neural network (CNN) and squeeze-and-excitation layers to detect lung and colon cancer from the histopathological images dataset (LC25000). Our proposed model achieved state-of-the-art accuracy in detecting cancerous cells and promises to bring this advancement to global healthcare for better medical diagnostics.

The rest of the paper is organized in the following order: Section 2 provides an insight into previous works that contribute to our achievement in this paper. Section 3 briefly overviews the techniques to construct our proposed model. Section 4 elaborates our CNN model architecture. Section 5 details the methods used to evaluate the results. Section 6 outlines the dataset and methodologies and reports the potential outcome of our research. Finally, Section 7 discusses the work that has been undertaken in this article as well as promising future work.

## 2. Related Work

### 2.1. Colon Cancer

Sena et al. [12] took a ’direct’ method, labeling raw photos rather than segmenting them in 2019. A total accuracy of 95% was reached, with most mislabeling related to a nearby category. Tests on an external dataset with a different resolution produced more than 80% accuracies. This study proved that a properly trained neural network may give fast, accurate, and reproducible labeling for colon cancer images, thereby improving the quality and timeliness of medical diagnostics. In 2019, Yoon et al. developed some improved systems based on the Visual Geometry Group (VGG), which won the classification task in the 2014 ImageNet Large Scale Visual Recognition Competition (ILSVRC), and performed two tests [13]. Firstly, they found the optimal modified VGG configuration for their incomplete dataset, yielding 82.50%, 87.50%, 87.50%, 91.40%, and 94.30% accuracies. And the second experiment used the best adjusted VGG configuration to assess the performance of the CNN model. Their proposed modified VGG-E configuration demonstrated the highest performance in terms of accuracy, loss, sensitivity, and specificity, achieving 93.48% accuracy, a loss of 0.4385, 95.10% sensitivity, and 92.76% specificity across the entire dataset. In a study in 2019, Kather et al. looked into whether deep convolutional neural networks (CNNs) might derive prognosticators directly from these widely available photos [14]. They manually identified single-tissue regions in 86 CRC tissue slides from 25 CRC patients, giving over 100,000 HE image patches, and utilized these to train a CNN using transfer learning, achieving an accuracy of more than 94%.

Wei et al. proposed a paper in 2020 where the prognostic analysis used histopathologic slides gathered from the Dartmouth-Hitchcock Medical Center in Lebanon, New Hampshire [15]. This dataset consisted of 326 slides for training, 157 for internal evaluation, and 25 for validation. The deep neural network had a mean accuracy of 93.5% (95% CI, 89.6–97.4%) in the internal evaluation of 157 slides compared to local pathologists’ accuracy of 91.4% (95% CI, 87.0–95.8%). For the external data collection, 238 slides for 179 different patients were received from 24 institutions in 13 states. The deep neural network attained an accuracy of 87.0% (95% CI, 82.7–91.3%), comparable to the accuracy of local pathologists of 86.6% (95% CI, 82.3–90.9%) on the external dataset. In 2020, Iizuka et al. trained convolutional neural networks (CNNs) and recurrent neural networks (RNNs) on biopsy histopathology whole-slide images (WSIs) from the stomach and colon [16]. The models were taught to categorize WSI as adenocarcinoma, adenoma, or non-neoplastic. They examined their models on three separate test sets, reaching AUCs of 0.96 and 0.99 for colonic cancer and adenoma, respectively. The results show that their models are generalizable and have considerable potential for use in a practical histopathological diagnostic workflow system. In the same year, Xu et al. introduced a deep learning-based technique for colorectal cancer identification and segmentation using digitized H&E-stained histology slides [17]. This study showed that the neural network approach achieved a median accuracy of 99.9% for normal slides and 94.8% for cancer slides when compared to pathologist-based diagnosis using H&E-stained slides digitized from clinical samples.

In 2021, Hamida et al. published research where they proposed two DL models using CNN-based histopathological image classification to diagnose colon cancer [18]. They achieved impressive patch-level classification results, with ResNet reaching a 96.98% accuracy rate. Their ResNet model was evaluated on CRC−5000, nct−crc−he−100k, and merged datasets and showed effectiveness with accuracy rates of 96.77%, 99.76%, and 99.98%, respectively. They evaluated these datasets with SegNet and achieved accuracy rates of 98.66%, 99.12%, and 78.39%, respectively. Researchers, including Babu and Tina, worked on automatically extracting high-level characteristics from colon biopsy images for automated patient diagnosis and prognosis using transfer learning architectures for colon cancer detection this year [19]. This study utilized a pre-trained CNN to extract visual features, which were then used to train a Bayesian optimal support vector machine classifier. Furthermore, this optimal network for colon cancer detection was examined using pre-trained neural networks, such as Inception-V3, VGG-16, and Alexnet. Additionally, four datasets were tested to assess the proposed framework: two were from Indian hospitals and were categorized as different magnifications (4×, 10×, 20×, and 40×), while the other two were public datasets of colon images. Based on public datasets analysis using the above-mentioned models, the Inception-V3 network achieved an accuracy range of 96.5% to 99% and outperformed the other tested frameworks. Tasnim et al. used CNN with pooling layers and MobileNetV2 models for colon cell image categorization [20]. The models were trained and tested at different epochs to determine the learning rate. The max pooling and average pooling layers were found to be 95.48% and 97.49% accurate, respectively. MobileNetV2 surpassed the other two models, with the highest accuracy of 99.67% and a data loss rate of 1.24.

Sakr et al. proposed a lightweight deep learning method in 2022, utilizing CNNs to efficiently detect colon cancer histopathological images and normalizing input before training [21]. The system achieved an accuracy of 99.50%, which was considered remarkable after comparative analysis with existing methods, highlighting its potential for improving colon cancer detection. Hasan et al. also used CNNs to analyze digital images of colon tissue to accurately classify adenocarcinomas in 2022 [22]. Automated AI diagnosis could accelerate assessments and reduce associated costs, leveraging modern DL and digital image processing techniques. The results showed accuracy rates of up to 99.80%, indicating that implementation of this approach could lead to automated systems for detecting various forms of colon cancer. This year, Talukder et al. introduced a hybrid ensemble feature extraction model aimed to efficiently detect colon cancer using machine learning and deep learning techniques [23]. Integrating deep feature extraction and ensemble learning with high-performance filtering for cancer image datasets, the computer-based model achieved impressive accuracy rates of 100% for colon cancer detection on the histopathological LC25000 dataset.

A study carried out by Bostanci’s research team in 2023 analyzed RNA-seq data from the extracellular vesicles of healthy individuals and colon cancer patients to develop predictive models for cancer presence and stage classification [24]. The study achieved high accuracy rates by utilizing both canonical machine learning and deep learning classifiers, including KNN, LMT, RT, RC, RF, 1-D CNN, LSTM, and BiLSTM. Canonical ML algorithms reached up to 97.33% accuracy for cancer prediction and 97.33% for cancer stage classification, while DL models achieved 97.67% and 98% accuracies, respectively. The results indicate that both ML and DL models can effectively predict and classify colon cancer stages, varying their performance depending on the number of features.

### 2.2. Lung Cancer

In 2019, Zhang et al. introduced a three-dimensional CNN that detects and classifies lung nodules as malignant or benign based on histological and laboratory results [25]. The well-trained model has a sensitivity of 84.4% (95% CI, 80.5–88.3%) and specificity of 83.0% (95% CI, 79.5–86.5%). Smaller nodules (<10 mm) have high sensitivity and specificity compared to bigger nodules (10–30 mm). Manual assessments from various doctor grades were compared to three-dimensional CNN results to validate the model. The results suggest that the CNN model outperformed the manual assessment. Pham et al. created a revolutionary two-step deep learning system to address the problem of false-positive prediction while retaining accurate cancer diagnosis [26]. Three hundred and forty-nine whole-slide lung cancer lymph node pictures were gathered, including 233 slides for training, 10 for validation, and 106 for testing. The first step was using a deep learning algorithm to exclude often misclassified noncancerous areas (lymphoid follicles). The second phase involved developing a deep-learning classifier to detect cancer cells. These two-step strategies decreased errors by 36.4% on average and up to 89% on slides containing reactive lymphoid follicles. Furthermore, 100% sensitivity was achieved in macro-metastases, micro-metastases, and isolated tumor cells.

Gertych et al. developed a pipeline that used a CNN and soft-voting as the decision function to identify solid, micro-papillary, acinar, and cribriform growth patterns, as well as non-tumor areas [27]. Slides from the main LAC were received from the Cedars-Sinai Medical Center (CSMC), the Military Institute of Medicine in Warsaw, and the TCGA portal. Several CNN models trained with 19,924 image tiles taken from 78 slides (MIMW and CSMC) were tested on 128 test slides from the three locations based on the F1-score and pathologist-manual tumor annotations. The best CNN produced F1-scores of 0.91 (solid), 0.76 (micropapillary), 0.74 (acinar), 0.6 (cribriform), and 0.96 (non-tumor), respectively. The overall accuracy in recognizing the five tissue classifications was 89.24 percent. Slide-based accuracy in the CSMC set (88.5%) was considerably higher (*p* < 2.3 $\times 10^{-4}$) than in the MIMW (84.2%) and TCGA (84%), indicating superior slide quality. Hatuwal and Thapa proposed a CNN to categorize an image as benign, adenocarcinoma, or squamous cell carcinoma in 2020 [28]. The model achieved 96.11% and 97.20% accuracies during training and validation, respectively. The model’s performance was evaluated using precision, F1-score, recall, and a confusion matrix.

Saif et al. sought to use and modify the current pre-trained CNN-based model to detect lung and colon cancer using histopathology pictures and improve augmentation strategies [29]. Eight distinct pre-trained CNN models were trained on the LC25000 dataset: VGG16, NASNetMobile, InceptionV3, InceptionResNetV2, ResNet50, Xception, MobileNet, and DenseNet169. The model’s performance was evaluated using precision, recall, F1-score, and accuracy. GradCAM and SmoothGrad were used to represent the pre-trained CNN models’ attention images that identify malignant and benign images. After training and testing on 1500 photos, the suggested model achieved an overall accuracy of 98.53%, whereas the VGG16 model achieved 96.67%. The proposed model had a sensitivity of 97.4% for adenocarcinoma, 99.6% for benign, and 98.6% for squamous cells. Abbas et al. used several off-the-shelf pre-trained (on ImageNet dataset) CNNs to classify the histopathological slides into three classes: lung benign tissue, squamous cell carcinoma, and adenocarcinoma [30]. The F1-scores of AlexNet, VGG-19, ResNet-18, ResNet-34, ResNet-50, and ResNet-101 on the test dataset showed results of 0.973, 0.997, 0.986, 0.992, 0.999, and 0.999, respectively. Srinidhi et al. created the first deep learning-based classifier to classify lung adenocarcinoma, lung squamous cell carcinoma, small cell lung carcinoma, pulmonary tuberculosis, organizing pneumonia, and normal lung in 2021 [31]. The EfficientNet-B5 model outperformed ResNet-50 and was chosen as the classifier’s backbone. Four medical centers tested 1067 slides with a classifier showing consistently high AUCs of 0.970, 0.918, 0.963, and 0.978. The intraclass correlation coefficients were greater than 0.873. In the same year, Han et al. used 50 top-ranked feature subset selection techniques for categorization [32]. The LDA (AUROC: 0.863; accuracy: 0.794) and SVM (AUROC: 0.863; accuracy: 0.792) classifiers, along with the $l_{2,1}$NR feature selection approach, performed optimally. Our investigation found that the random forest (RF) classifier (AUROC: 0.824; accuracy: 0.775) and the $l_{2,1}$NR feature selection approach (AUROC: 0.815; accuracy: 0.764) performed well on average. Furthermore, the VGG16 DL algorithm (AUROC: 0.903; accuracy: 0.841) beat all other machine learning methods when combined with radiomics.

In a work in 2021, P Marentakis et al. wanted to look at the potential of NSCLC histological classification into AC and SCC using various feature extraction and classification approaches on pre-treatment CT scans [33]. The picture dataset used (102 patients) was obtained from the publicly available cancer imaging archive collection (TCIA). They looked at four different technique families: (a) radiomics with two classifiers (kNN and SVM), (b) four cutting-edge CNNs with transfer learning and fine tuning (Alexnet, ResNet101, Inceptionv3, and InceptionResnetv2), (c) a CNN combined with a long short-term memory (LSTM) network to fuse information about the spatial coherency of tumor CT slices, and (d) combinatorial models (LSTM + CNN + radiomics). Additionally, two qualified radiologists independently assessed the CT pictures. Our findings indicated that Inception was the best CNN (accuracy = 0.67, auc = 0.74). LSTM + Inception outperformed all other algorithms (accuracy = 0.74, auc = 0.78). Additionally, LSTM + Inception beat experts by 7–25% (*p* < 0.05).

Abdul Rahaman Wahab Sait developed a deep-learning model for lung cancer detection using PET/CT images comprising 31,562 annotated images in 2022 [34]. He addressed challenges like computational complexity by employing techniques such as preprocessing, augmentation, and model optimization. CNN-based DenseNet-121 and MobileNetV3 models were constructed to extract features and identify the types of lung cancer. His model achieved a high accuracy of 97.5% and a Cohen’s Kappa value of 95.8 with fewer parameters and can potentially aid in early-stage lung cancer detection. In 2022, Shandilya and Nayak formulated a computer-aided diagnostic (CAD) approach for classifying histopathological images of lung tissues [35]. Utilizing a publicly available dataset of 15,000 samples of histopathological photographs, they extracted image features and assessed seven pre-trained convolutional neural network models, including MobileNEt, VGG-19, ResNet-101, DenseNet-121, DenseNet-169, InceptionV3, Inception ResNet-V2, and MobileNetV2 for histopathological images classification of 15,000 samples. Among them, ResNet-101 attained the highest accuracy of 98.67%. In the same year, Ameer et al. developed a deep learning model for automated lung cancer cell detection in histopathological tissue images [36]. They used several models encompassing InceptionV3, Random Forest, and CNNs. These models were trained meticulously to extract important features from the images, thereby improving the efficiency and accuracy of lung cancer cell detection. The proposed model achieved remarkable accuracy of 97.09%, precision of 96.89%, recall of 97.31%, F1-score of 97.09%, and specificity measures of 96.88%.

In 2023, Priyadarsini et al. proposed a framework designed to detect and categorize lung cancer using deep learning models trained on X-ray and CT scan images [37]. Three deep learning models—sequential, functional, and transfer models—were implemented and trained on open-source datasets to improve patient treatment. Emphasizing deep learning methods, particularly CNNs, they extracted specific features from image datasets. The functional model stood out with 99.9% accuracy and 99.89% specificity for lung cancer detection while requiring fewer parameters and computational resources than existing models. Siddiqui et al. introduced a pioneering method for lung CT image classification, focusing on enhancing efficiency and accuracy in 2023 [38]. The method employed an enhanced Gabor filter for preprocessing, reducing parameters using the Gauss–Kuzmin distribution to maintain detail while minimizing computational load. Feature selection was conducted via an enhanced deep belief network (E-DBN) with two cascaded restricted Boltzmann machines (RBMs), followed by evaluation with five classifiers, leading to the selection of a support vector machine (SVM) for optimal performance. The experimental results demonstrated superior accuracy and sensitivity compared to existing methods, with the proposed approach achieving an F1-score of 99.37% and accuracy of 99.424%. These findings suggest promising advancements in lung cancer diagnosis through advanced image processing techniques. Wahid et al. proposed a CAD in 2023 utilizing CNNs to detect lung cancer within the LC25000 dataset, encompassing 25,000 histopathological color image samples [39]. Four CNN models, including ShuffleNet-V2, GoogLeNet, ResNet-18, and a customized CNN model, were used. Among them, ShuffleNet-V2 achieved the highest accuracy of 99.87% and exhibited the shortest training time of 1202.3 s.

### 2.3. Lung and Colon Cancer

A study by Masud et al. sought to offer a computer-aided diagnosis system for diagnosing squamous cell carcinomas, lung adenocarcinomas, and colon adenocarcinomas using convolutional neural networks and digital pathology pictures in 2020 [40]. A shallow neural network design was employed to identify the histological slides as squamous cell carcinomas, adenocarcinomas, or benign lung. A similar methodology was used to classify adenocarcinomas and benign colon tumors. The diagnosis accuracy for the lung and colon was around 97% and 96%, respectively. Garg et al. also published a work in 2020 that sought to use and modify the current pre-trained CNN-based model to detect lung and colon cancer using histopathology pictures and improved augmentation strategies [41]. This article trained eight distinct pre-trained CNN models on the LC25000 dataset: VGG16, NASNetMobile, InceptionV3, InceptionResNetV2, ResNet50, Xception, MobileNet, and DenseNet169. The model’s performance was evaluated using precision, recall, F1-score, accuracy, and AUROC scores. The results show that all eight models achieved significant outcomes, ranging from 96% to 100% accuracy. GradCAM and SmoothGrad were then utilized to represent the attention images of pre-trained CNN models that identify malignant and benign images.

Ali et al. presented a novel multi-input dual-stream capsule network in 2021 that used the powerful feature learning capabilities of conventional and separable convolutional layers to classify histopathological images of lung and colon cancer into five categories (three malignant and two benign) [42]. They preprocessed the dataset using a novel color balancing technique that attempts to adjust three color channels before gamma correction and sharpening the most noticeable features. The suggested model was given two inputs simultaneously (one with original photos and the other with preprocessed images), allowing it to learn features more effectively. The provided findings reveal that the model had an overall accuracy of 99.58% and an F1-score of 99.04%.

In research published in 2021, Mehedi et al. described a unique DL-based supervised learning approach that uses pathological image analysis to identify five distinct tissue types (two noncancerous, three cancerous) present in lung and colon tumors [40]. The LC25000 dataset was utilized for both training and validation techniques. Two different kinds of domain transformations were used to obtain four sets of features. The resulting features were concatenated to create a combined collection of features with both kinds of information. The results confirm that the model is accurate and reliable (96.38% F1-score) for identifying lung and colon cancer, with a peak classification accuracy of 96.33%.

In 2022, Hage et al. developed CADs using artificial intelligence to accurately classify different types of colon and lung tissues based on histopathological images [43]. The researchers utilized machine learning models, including XGBoost, SVM, RF, LDA, MLP, and LightGBM, to classify histopathological images that they obtained from the LC25000 dataset. The results showed that the models achieved satisfactory accuracy and precision in identifying lung and colon cancer subtypes, among which the XGBoost model performed the best, with an accuracy of 99% and an F1-score of 98.8%. Talukder et al. developed a hybrid ensemble model for the efficient detection of lung and colon cancer, which combined deep feature extraction and ensemble learning techniques to analyze histopathological image datasets using a set of metrics (LC25000) [23]. The model was evaluated using high-performance filtering and achieved high accuracy rates for detecting lung and colon cancer of 99.30%. Mehmood et al. also developed a highly accurate and computationally efficient model for the rapid and precise diagnosis of lung and colon cancer in 2022 [44]. They utilized a dataset consisting of 25,000 images divided into five classes. To train the model, they modified four layers of the pre-trained neural network, AlexNet, and achieved an overall accuracy of 89%. They further enhanced the image quality through contrast enhancement techniques, resulting in an improved accuracy of 98.4%.

In 2023, Singh et al. presented an ensemble classifier that combined random forest, support vector machine (SVM), and logistic regression [45]. The deep features from lung and colon cancer images, obtained from the LC25000 dataset, were extracted using VGG16 and binary pattern methods. These methods yielded the initial relevant features for the ensemble classifier. The proposed methodology achieved an average accuracy of 99%, precision of 99%, and recall of 98.8%. Bhattacharya et al. proposed a framework that combined deep learning and meta-heuristic approaches for the accurate prediction of lung and colon cancer from histopathological images in which they trained deep learning models, ResNet-18 and EfficientNet-b4-wide, on the LC25000 dataset and extracted deep features [46]. They developed the AdBet-WOA hybrid meta-heuristic optimization algorithm to remove redundancy in the feature vector. They used the SVM classifier to distinguish lung and colon cancer, achieving an impressive accuracy of 99.96%. Al-Jabbar et al. developed three strategies, each with two systems, to analyze the dataset in 2023 [47]. The GoogLeNet and VGG-19 models were used to enhance the images and increase the contrast of affected areas, followed by dimensionality reduction using the PCA method to retain essential features. They used ANN with fusion features of CNN models and handcrafted models and reached a high sensitivity of 99.85%, precision of 100%, accuracy of 99.64%, specificity of 100%, and AUC value of 99.86%, indicating the effectiveness of the proposed approach for the early diagnosis of lung and colon cancer.

## 3. Background Work

### 3.1. Residual 1D Convolution Networks

**Figure 1:**
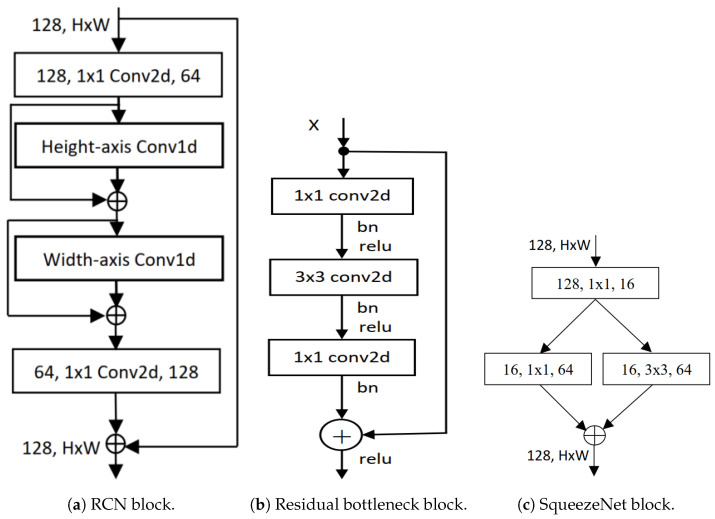
Illustration of block diagrams found in (**a**) Residual network [50], (**b**) Residual 1D convolutional network [48], and (**c**) SqueezeNet [49].

Residual 1D convolution networks (RCNs), as shown in Figure 1a, are a technique introduced by Shahadat and Maida where 2D convolution operation is replaced by 1D CNN layers with residual connections [48]. Residual bottleneck blocks in this particular network type focus on learning residual functions rather than full transformations, as shown in Figure 1b. They operate over one-dimensional data, such as a time-series signal, where the convolutional filter moves along the time axis. Its architecture comprises an input layer for 1D sequential data, convolutional layers with activation functions, residual connections for skip connections, a pooling layer for downsampling the sequence, and fully connected layers for final classification. The 1D CNN layer processes 1D input at a time (X∈HorX∈W) whereas the 2D CNN layer takes (X∈H×W). This way, the input cost becomes 2H instead of $H^{2}$, and the operation is explained by the equation given as,

(1) $$C_{O_{\left(i,n\right)}}=\sum_{a\in N_{k}\left(i\right)}W_{a,n}X_{i+a-1,n}$$

where the neighborhood of pixel *i* with a spatial extent of *k* is $N_{k}\in R^{k\times d_{in}}$, and the trainable weight $W\in R^{k\times d_{out}\times d_{in}}$ is the shared weight to compute the output for all pixel positions *i*. Additionally, the n-th channel of the trainable weight *W* is applied to the n-th channel of the input *X* in order to generate the n-th channel of the output feature map $C_{O}$, where the computational cost is calculated as,

(2) $$Cost_{1D}=h\cdot d_{out}\cdot k$$

The total cost is multiplied by 2 for the two 1D CNN layers. This type of network is used to mitigate the vanishing gradient problem, making it easier to train deep networks and helping in learning identity mappings.

### 3.2. Squeeze-And-Excitation Networks

The squeeze-and-excitation network (SENet) is designed to improve CNNs by capturing channel interdependencies with minimal computational overhead [49]. SENet, depicted in Figure 1c, introduces parameters to each channel within a convolutional block, enabling the network to adaptively adjust the weighting of each feature map. The network gives equal weight to each channel when generating the output feature maps. It consists of two main operations named as “squeeze” and “excitation”. During the squeeze operation, the spatial dimensions of the input feature maps are reduced while retaining the channel-wise information. This process involves generating a channel descriptor and usually includes global pooling operations. On the other hand, the excitation operation utilizes the channel descriptor to calculate channel-wise scaling factors that determine how much emphasis should be placed on each channel.

The factors are computed using a small neural network, such as ReLU or sigmoid. It can be constructed for any transformation as,

(3) $$F_{\mathrm{tr}}:X\to U,\;X\in R^{H^{\prime }\times W^{\prime }\times C^{\prime }},\;U\in R^{H\times W\times C}$$

where $F_{\mathrm{tr}}$ is a convolutional operator. If we suppose that $V=[v_{1},v_{2},\ldots ,v_{C}]$ is a set of learned filter channels, then we can write the outputs of $F_{\mathrm{tr}}$ as $U=[u_{1},u_{2},\ldots ,u_{C}]$, where

(4) $$u_{c}=v_{c}*X=\sum_{s=1}^{C^{\prime }}v_{c}^{s}*x_{s}.$$

**Figure 2:**
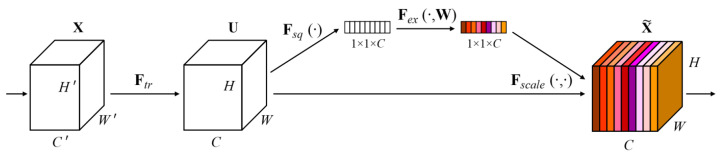
SqueezeNet block [49].

In the equation above, ∗ represents convolution. $v_{c}^{s}$ is a 2D spatial kernel. It acts as a single channel of $v_{c}$, which operates on the corresponding channel of *X*. The output is the result of a summation across all channels, encompassing channel dependencies within $v_{c}$. However, these dependencies are intertwined with the spatial correlation captured by the filters. The expanded diagram of SENet is shown in Figure 2.

### 3.3. SqueezeNext Architecture

**Figure 3:**
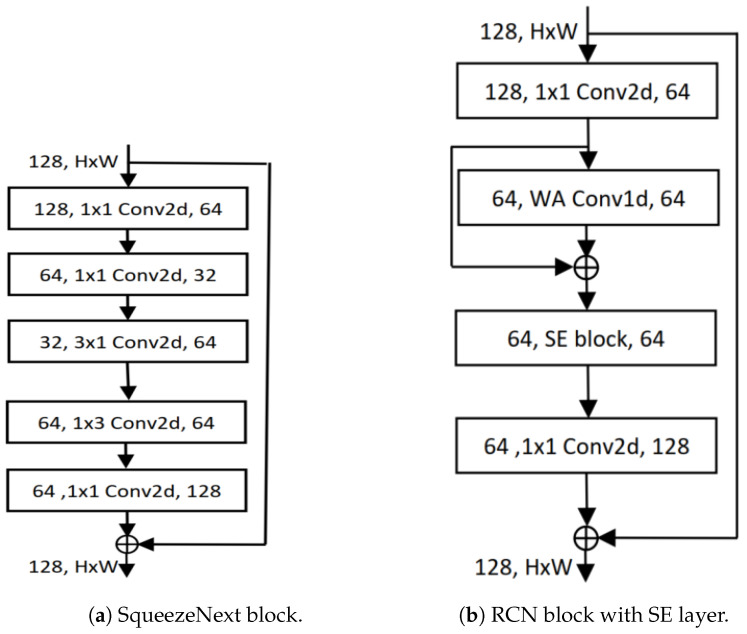
Illustration of block diagrams found in (**a**) SqueezeNext [51], and (**b**) SEC [52].

**Figure 4:**
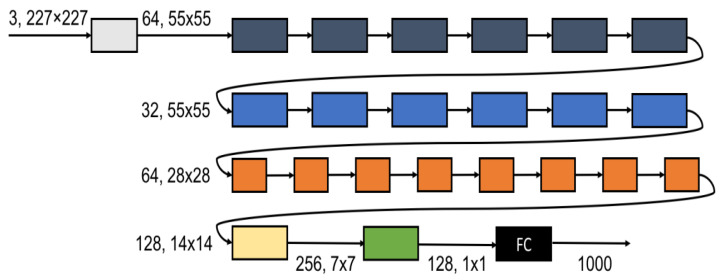
SqueezeNext network architecture (23 layers) [51].

The SqueezeNext architecture is a compact network designed to be trained with few model parameters from the beginning, rather than relying on compression methods to reduce the parameter count. One of the methods used by the SqueezeNext architecture to implement this efficiently is utilizing low-rank filters. Assuming that the input to the *i*-th layer of the network with K×K convolution filters is $X\in R^{H\times W\times d_{in}}$, the output activation of $Y\in R^{H\times W\times d_{out}}$ is produced. This layer transformation consumes $K^{2}\cdot d_{in}\cdot d_{out}$ cost and the filters consist of $d_{out}$ tensors of size $K\times K\times d_{in}$. The goal is to reduce the parameters, *W*, using a low-rank basis, $\tilde{W}$, in post-training compression. However, upon examining the trained network weights, it becomes evident that they usually do not exhibit a low-rank structure. Therefore, many networks necessitate some form of retraining. Instead of doing that, redesigning the network using the low-rank decomposition from the outset encourages the network to learn a low-rank structure from the beginning. This is the strategy adopted by the SqueezeNext architecture. Firstly, the *K*-convolutions are decomposed into two separable convolutions of size 1×K and K×1, which reduces the number of parameters from $K^{2}$ to 2K and also increases the network’s depth. Both of these convolutions have a ReLU activation and a batch normalization layer [51]. The block diagram is depicted in Figure 3a. This SqueezeNext block is stacked together to construct the SqueezeNext network architecture; a 23-layer network architecture is depicted in Figure 4.

### 3.4. Residual 1D Block with SE Layer

Introduced by Shahadat, “Squeeze-and-Excitation-based 1D Convolutional Networks” (SECs) represent a parameter-efficient, mobile-embedded deep learning architecture. The SEC architecture, shown in Figure 3b, replaces the 1D CNN layer with a squeeze-and-excitation (SE) block to enhance cost efficiency and reduce computational complexity [52]. As the SEC replaces an RCN layer in RCNs, the cost reduction is directly analyzed between these two layers.

The cost comparison between the computational costs of the residual 1D CNN and the SE block is expressed as:

(5) $$Cost_{R}=\frac{{\mathrm{Cos}t}_{1D\;\mathrm{CNN}}}{{\mathrm{Cos}t}_{\mathrm{SE}-\mathrm{layer}}}=\frac{h\cdot d_{in}\cdot k}{d_{in}\cdot d_{out}}=\frac{h\cdot k}{d_{out}}$$

which equals $\frac{h\cdot k}{d_{out}}$, where $Cost_{R}$ is the ratio comparing the computational costs between the original residual 1D convolutional layer and the SE block. Also, *k* is the kernel, $d_{in}$, and $d_{out}$ denote the number of input and output channels.

### 3.5. Reduced CNN Layer Network

**Figure 5:**
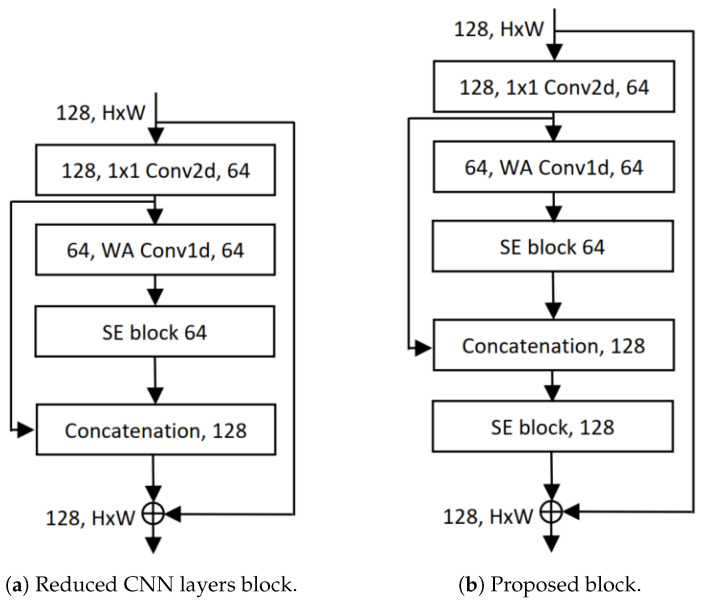
Illustration of block diagrams found in (**a**) Reduced CNN layers Network [48,53,54], (**b**) our proposed network architectures.

A reduced CNN layer network functions similarly to traditional CNNs, achieving comparable performance while minimizing computational cost and model size [53]. The bottleneck, SENets, and channel squeezing are network architectures that utilize reduced CNN layers. The fundamental architecture of ResNet includes the basic residual block, which consists of two 3×3 convolutional layers and a residual connection. A bottleneck residual block also incorporates a 1×1 convolution, depicted in Figure 1b. The computational cost of the basic block is twice the cost of the spatial CNN layer, which is $2\cdot h^{2}\cdot d_{in}\cdot d_{out}\cdot k^{2}$. Another essential component of the ResNet architecture is the bottleneck layer, which includes two pointwise convolution layers: a first, known as the ConvDown layer, and a final, known as the ConvUp layer. The first layer reduces $d_{in}$, passed through the spatial convolution layer, whereas the final layer is responsible for increasing $d_{out}$ of the spatial CNN layer. In the reduced CNN block, this ConvUp layer in SEC [52] is replaced by the channel concatenation layer shown in Figure 5a.

## 4. Proposed Architecture

The proposed parameter-efficient architecture is a novel, lightweight, mobile-embedded network designed for the accurate detection of lung and colon cancer subtypes using histopathological images. The primary objective of this architecture is to reduce computational costs while maintaining high accuracy, making it suitable for deployment on mobile devices. We utilize a combination of residual 1D convolutional neural networks (Conv1D) [48] and squeeze-and-excitation (SE) [49] blocks as their fundamental building blocks and construct our proposed block architecture, depicted in Figure 5b. Unlike traditional 2D CNNs, which are computationally expensive, the proposed architecture employs 1D CNNs along the width axis, called residual 1D CNN (RCN) [48]. SE blocks are integrated into the architecture to implement a channel-wise attention mechanism. This mechanism allows the network to selectively emphasize important channels and suppress less relevant ones, improving feature representation and overall efficiency.

Regarding computing, our suggested architecture is more economical than the lightweight SqueezeNext block [51]. We only used one instead of two pointwise CNN layers in the SqueezeNext block. Savings of at least $h\times w\times d_{in}\times d_{out}$ costs are beneficial. We use the RCN and SE layers to replace the two separable CNN layers (3×1 and 1×3). These changes’ cost comparisons are described as follows:

(6) $$\begin{array}{cc} Cost_{R} & =\frac{Cost\;of\;3\times 1\;\mathrm{CNN}}{Cost\;of\;\mathrm{RCN}}+\frac{Cost\;of\;1\times 3\;\mathrm{CNN}}{Cost\;of\;\mathrm{SE}-Block}\\ & =\frac{h\cdot w\cdot d_{in}\cdot d_{out}\cdot k}{w\cdot d_{out}\cdot k}+\frac{h\cdot w\cdot d_{in}\cdot d_{out}\cdot k}{d_{in}\cdot d_{out}}=h\cdot d_{in}+h\cdot w\cdot k \end{array}$$

where the number of input channels, height, width, and output height are represented by the variables $d_{in}$, *h*, *w*, and $d_{out}$, the kernel size is *k*. Equation (6) shows that our proposed block is $h\cdot d_{in}+h\cdot w\cdot k$ times more cost-effective than the separable CNN layers in the SqueezeNext block.

Our modifications are not limited to these. To decrease the complexity of the network, we also replace the ConvUp layer (the 1×1 CNN layer is used to increase the number of output channels) using channel concatenation. The absence of the channel-based weight layer from our channel concatenation results in decreased performance attributed to the pointwise CNN layer. We employ the SE layer, which helps to improve performance by utilizing a channel-wise attention method, to get around this restriction. In addition, this SE layer is less expensive than the 1×1 CNN layer, which is described as,

(7) $$Cost_{R}=\frac{Cost\;of\;1\times 1\;\mathrm{CNN}}{Cost\;of\;\mathrm{SE}-Block}=\frac{h\cdot w\cdot d_{in}\cdot d_{out}}{d_{in}\cdot d_{out}}=h\cdot w$$

The above equation describes the computational cost reductions by our proposed block compared to the SqueezeNext block with a factor of $Cost_{R}=h\cdot w\cdot d_{in}\cdot d_{out}+h\cdot d_{in}+h\cdot w\cdot k+h\cdot w$. So, our proposed architecture is more parameter-efficient and cost-effective than the well-known compact SqueezeNext block.

We utilize precisely two SE layers to boost performance. The SE layers take output feature maps from the RCN layer as input and produce better output feature maps using channel-wise feature recalibration. These channel-wise feature recalibrations improve the model’s performance, reduce overfitting, and focus on important channels. We stack this proposed block in the SqueezeNext network architecture to construct the proposed network.

## 5. Performance Evaluation

Evaluating performance is crucial for assessing how accurately a model predicts outcomes. It confirms that the model fits the training data well and is also effective for new and unseen data. Common evaluation metrics include accuracy, precision, recall (sensitivity), and the F1-score.

### 5.1. Accuracy

Accuracy is a crucial performance evaluation metric used in machine learning and statistics for classifying problems. It measures the correctness of the trained parameters or cases and assesses the proportion of correct observations among the total observations. The accuracy is calculated as,

(8) $$Accuracy=\frac{TP+TN}{TP+FP+TN+FN}$$

where *TP* is true positives, which is the number of correctly predicted positive instances by the model. For example, if a person has the disease, the test is positive; *TN* stands for true negatives, which represents the number of negative instances correctly predicted by the model. This means that if the person does not have the disease, the test results are negative; *FP* stands for false positives, which is the number of negative instances incorrectly predicted as positive by the given model. This means that the test can show a positive result even if the person is not diseased. Lastly, *FN* stands for false negatives, representing the number of positive instances that the model incorrectly predicts as negative. This means that even if a person is diseased, the test results are negative. When the numbers of TP and TN are high compared to the total predictions, the accuracy is high. On the other hand, if the numbers of TP and TN are low compared to the total predictions, the accuracy is low. The total prediction is the sum of all the predictions: TP + TN + FP + FN. Therefore, we can conclude that higher accuracy is needed to enhance the overall reliability of the model’s predictions. By improving accuracy, we can decrease the occurrences of FP and FN, leading to more reliable and effective decision-making.

### 5.2. Precision

Another way to measure the performance of machine learning models is using the precision, which is calculated as the ratio of true positives to the sum of true positives and false positives. The equation to calculate the precision is defined as,

(9) $$Precision=\frac{TP}{TP+FP}$$

In the equation, a high precision value indicates that when the model predicts a positive outcome, it is usually correct. This suggests that the model has a low number of false positives, making its positive predictions reliable. Conversely, a low precision value indicates that the positive predictions made by the model are incorrect. Therefore, we can conclude that low precision can have a negative impact.

### 5.3. Recall

Recall, also known as sensitivity, is another important metric used to assess the performance of machine learning models. It quantifies the model’s capability to accurately predict all the positive instances in a dataset. It is calculated as the ratio of true positives to the sum of true positives and false negatives. The formula to calculate recall is as follows,

(10) $$Recall=\frac{TP}{TP+FN}$$

Based on the above equation, we understand that a high recall signifies that the model predicts most actual positives, while a low recall score indicates a high number of false negatives, leading to the model failing to predict actual positives. A low recall score could result in significant issues, particularly when conducting disease screenings, and can lead to severe repercussions.

### 5.4. F1-Score

In the statistical analysis of binary classification, the F1-score is used to measure predictive performance. It is calculated as the ratio of two times precision and recall (both determined using the previously mentioned equations) to the sum of precision and recall. The F1-score can also be defined as the harmonic mean of precision and recall. Its values range from 0 to 1, where 0 indicates the lowest performance and 1 indicates the highest performance.

(11) $$F1-Score=\frac{2\times Precision\times Recall}{Precision+Recall}=\frac{2\times TP}{2\times TP+FP+FN}$$

Based on the equation above, a high F1-score indicates that the model identifies a greater proportion of positive instances while minimizing false positives. Conversely, a low F1-score suggests that the model struggles to accurately predict positive instances.

## 6. Experimental Results

### 6.1. Dataset Description

**Figure 6:**
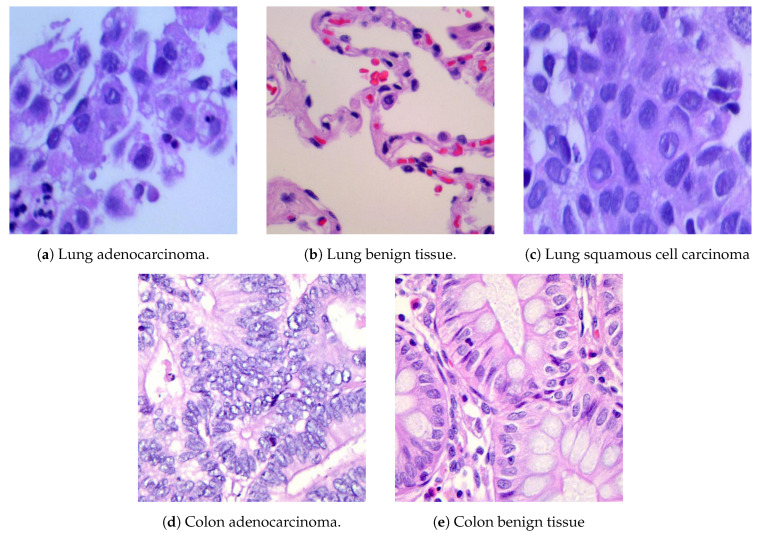
Randomly selected lung and colon cancer histopathological images from the LC25000 dataset [55].

This research uses the lung and colon cancer histopathological images LC25000 dataset [55]. This dataset comprises two main categories of cancer cells: colon adenocarcinoma and benign colon tissue, and lung adenocarcinoma, lung squamous cell carcinoma, and benign lung tissue. The sample images of these lung and colon cancer categories are depicted in Figure 6.

They contain 25,000 color cancer cell images from five classes of lung and colon cancer’s benign and malignant tissue images. Initially, 1250 photos were taken from cancer tissues on pathology glass slides at the James A. Haley Veterans’ Hospital in Tampa, Florida, with 250 images for each category [40]. The original LC25000 dataset includes 750 lung tissue samples, comprising 250 adenocarcinoma, 250 squamous cell carcinomas, and 250 benign tissue samples. The dataset includes 500 colon tissue samples, with 250 adenocarcinoma and 250 benign tissue samples. These photos were then augmented with techniques like rotation and flipping, resulting in a collection of 5000 images per class and totaling 25000 images for lung and colon cancers.

The photos were originally 1024 × 768 pixels but were cropped to 768 × 768 pixels before augmentation to retain a uniform square shape. All photos are HIPAA-compliant, vetted, and freely available to AI researchers, making them an invaluable resource for creating more effective diagnostic tools. In our experimental analysis, we resized all the images to 256 × 256 pixels and randomly cropped them to 224 × 224 pixels. After resizing, we normalized the images using their mean and standard deviation. Moreover, we split the main dataset (LC25000) into two parts: 80% for training and 20% for testing samples. The image distribution of the lung and colon dataset is explained in Table 1.

### 6.2. Methodology

Similar hyperparameters were used to the original SqueezeNet architectures [51]. We then examined 23-layer architectures with the block multipliers “[6, 6, 8, 1]”. Likewise, we constructed 44-layer architectures with the block multipliers “[12, 12, 16, 2]”. All the proposed architectures were trained using various batch sizes, including 8, 16, 32, 64, and 128.

The LC25000 dataset contains 25,000 images resized and cropped to 224 × 224 pixels. Mean/std normalization was applied to preprocess our image data. All models were trained using the stochastic gradient descent (SGD) optimizer. We applied warmed-up linear learning for the first ten epochs, followed by cosine learning scheduling from epochs 11 to 120.

### 6.3. Results Analysis

This section evaluates the outcomes of using our proposed model. It is important to note that the accuracy does not significantly improve or decline as the batch sizes increase. Additionally, our model showed excellent performance for a smaller number of epochs. Furthermore, our model showed exceptional accuracy in colon cancer detection and achieved nearly perfect results in all tests. It takes fewer epochs to show state-of-the-art performance than the lung cancer detection using our model. The different batch sizes effectively train our models, demonstrating that our model can process varying amounts of input data without sacrificing effectiveness. Finally, Table 2 and Table 3 present an overview of our results, indicating that our model achieved the best performance for all datasets with a fixed batch size of 64.

### 6.4. Results Comparisons

Table 4, Table 5, Table 6 and Table 7 compare our proposed method and several previous well-known studies and relevant network architectures. Table 4 compares several models and demonstrates the direct effectiveness of our modified architecture. It is crucial to note that these studies are based on using different datasets and imaging with different numbers of epochs, and batch sizes, which makes direct comparison a bit more difficult. However, our proposed method achieves perfect scores in all evaluation metrics, i.e., 100% accuracy rate, precision, sensitivity, and F1-score, showcasing its versatility in detecting lung and colon cancer. This comparison also offers a contextual understanding of our proposed model in relation to other methodologies.

#### 6.4.1. Lung Cancer

Here, we discuss the comparison of lung cancer detection between various published works and our proposed approach. Our mobile-supported and parameter-efficient method, a 1D convolutional neural network with an SE layer, outperforms all of the other methods as seen in Table 6. Our method utilized CT scan imaging of the publicly available LC25000 dataset and achieved perfect metrics, i.e., accuracy rate, precision, recall, and an F1-score of 100%. Remarkably, despite using a different dataset from LIDC-IDRI and a different network known as AlexNet and GoogLeNet, the method proposed by Vinod Kumar and Brijesh Bakariya [121] also achieved the same precision score as ours using computer-based models. Nonetheless, our achievements in terms of accuracy exceed all of the previous work, as detailed in Table 6.

#### 6.4.2. Colon Cancer

This section portrays the comparative efficacy of various methodologies in detecting colon cancer, as enumerated in Table 5. Utilizing CT scan imaging from the LC25000 dataset, our 1D convolutional network with SE layers achieves a 100% accuracy rate, precision, recall, and F1-score in the detection of colon cancer. Our proposed method surpasses nearly all studies referenced in Table 5, demonstrating the model’s capability to identify all instances of colon cancer presence or absence across both trained and new datasets. Additionally, our model maintains consistent performance across 30 epochs and 64 batch sizes, with 0.35 million parameters. It is worth noting that other computer-based models, such as the CNN model described by Dabass et al. (2022) [87], also achieved 100% accuracy scores using a computer-based deep learning model.

#### 6.4.3. Lung and Colon Cancer

We compared lung and colon cancer detection among various published methods as presented in Table 7. The proposed method, 1D CNN with squeeze-and-excitation (SE) layers, used CT scans imaging from the publicly available LC25000 dataset as the input. The 1D CNN with SE layers is trained on this data. The model identifies features related to the presence of lung and colon cancer. Our proposed method achieved a remarkable 100% accuracy rate, precision, recall, and F1-score in lung and colon cancer detection. This high accuracy indicates that the proposed model consistently correctly identifies all the instances of colon and lung cancer present or absent in the dataset. It consistently performs well over 50 epochs with a batch size of 64, using 0.36 million parameters. In machine learning, “epochs” refers to the number of times the entire dataset is passed through the model during training. On the other hand, “batch size” refers to the number of samples processed before the model’s internal parameters are updated. This means the model can be trained on various datasets, not just a specific set. Additionally, it outperforms almost all the other methods mentioned in Table 7. Additionally, the LightGBM computer-based model [136] proposed by Indu Chhillar and Ajmer Singh in 2024 also achieved the same accuracy as our model using CT scan imaging from the LC25000 dataset [136].

### 6.5. Final Remarks

**Figure 7:**
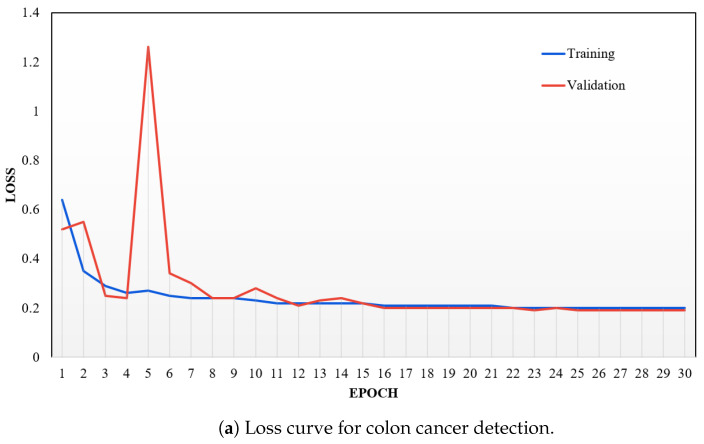

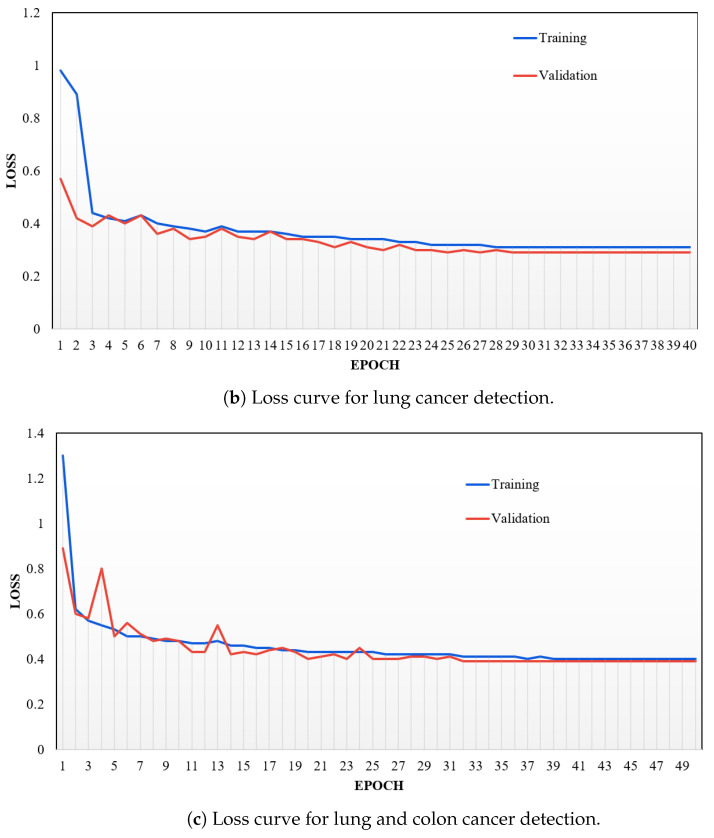
Training vs. validation loss diagrams to analyze overfitting in our proposed model across different numbers of epochs.

Our model outperformed existing models significantly, which emphasizes the efficiency of our approach. Surprisingly, our model accomplished 100% accuracy in detecting colon cancer across all batch sizes. It also achieved 100% accuracy in detecting lung cancer. It achieved perfect scores in detecting lung and colon cancer types with a batch size of 64, while maintaining consistently high results in other areas. While existing models have attained comparable performance, none have consistently reached the best accuracy across three cancer types as our model has. Despite implementing the same SqueezeNet network as the studies by Mohamed et al. (2023) [91] and Suominen et al. (2024) [134], our models achieved higher accuracy rates. Moreover, this suggests that combining SE layers and 1D convolutional networks can effectively enhance feature extraction capabilities and achieve state-of-the-art performance in medical image analysis where timely and accurate results are crucial. Our model achieved 100% accuracy for lung cancer, colon cancer, and both lung and colon cancers. We analyzed the overfitting of our model by examining the training and validation loss, as shown in Figure 7a–c. This analysis was performed for each epoch across all categories of lung and colon cancer. Our findings indicate that the model does not overfit on the datasets for lung cancer, colon cancer, and the combined lung and colon cancer images.

To create a successful machine learning (ML) model, focusing on the network’s ability to generalize and its reliability is essential. A large and diverse dataset is needed for effective training, so we used data augmentation to artificially expand the dataset by making small changes to the original data. This expansion allowed our model to recognize and generalize from a wider range of patterns and abnormal details in histopathological images. It significantly contributed to the model’s performance across different batches and resulted in the highest accuracy. Additionally, it helped us overcome overfitting (as shown in Figure 7a–c), which is crucial for learning detailed features of cancerous diseases without being misled by identical patterns.

## 7. Conclusions and Future Work

According to the World Health Organization (WHO), lung and colon cancer were the leading causes of death in 2020 [137]. Early diagnosis is crucial to overcome this issue. A study, proposed by a CNN network, constructed with 1D convolutional networks and SE layers to detect lung and colon cancer features from the large LC25000 dataset. Historically, diagnosis was a complex and lengthy process. The aim was to propose an approach that is not only efficient but also computationally economical by minimizing parameters; in this case, only 0.35 M parameters were used. Overall, we achieved a 100% accuracy, precision, recall, and F1-score across various batch sizes and epochs, indicating significant progress and the reliability of our model.

However, the limitation of this work is that the model has only been applied to the classification of lung and colon cancers, even though it shows potential for detecting them. Further analysis of the results indicates that there is still room for improvement in detecting other types of cancer in order to achieve optimal performance. Our comparisons show that our method outperforms almost all previous studies. Implementing this mobile-supported identification method in healthcare will assist pathologists in diagnosing lung and colon cancer more easily and reliably. In the future, we plan to apply our diagnostic method on other medical disease detection datasets to expand the scope of our study and improve the accuracy rate. This will enable us to extend our contributions to the detection of other types of cancer.

## Figures and Tables

**Table 1:** Distributions of lung and colon cancer LC25000 histopathological images dataset.

Data Samples	Lung Dataset			Colon Dataset		Total
	Adenocarcinoma	Cell Carcinomas	Benign	Adenocarcinoma	Benign	
Training Data Samples	4000	4000	4000	4000	4000	20,000
Testing Data Samples	1000	1000	1000	1000	1000	5000

**Table 2:** The performance on the LC25000 dataset to detect lung and colon cancer using our proposed networks.

Dataset	Epochs	Parameters	Batch Size	Testing Accuracy
Colon Cancer	30	0.35M	8	100
			16	100
			32	100
			64	100
			128	100
Lung Cancer	40	0.35M	8	99.17
			16	100
			32	100
			64	100
			128	100
Lung and Colon Cancer	50	0.36M	8	99.6
			16	99.94
			32	99.98
			64	100
			128	99.98

**Table 3:** The overall performance on the LC25000 dataset using our proposed networks.

Dataset	Epochs	Parameters	Batch Size	Accuracy	Precision	Recall	F1-Score
Colon Cancer	30	0.35M	64	100%	100%	100%	100%
Lung Cancer	40	0.35M	64	100%	100%	100%	100%
Lung and Colon Cancer	50	0.36M	64	100%	100%	100%	100%

**Table 4:** The performance on the LC25000 dataset to detect lung and colon cancer using some relevant networks and our proposed network [56].

Dataset	Model	Epochs	Parameters	Testing Accuracy
Colon Cancer	RCN	30	0.365M	99.69
	SEC		0.36M	99.77
	Reduced CNN		0.35M	99.91
	Our proposed model		0.35M	100
Lung Cancer	RCN	40	0.365M	99.65
	SEC		0.36M	99.69
	Reduced CNN		0.35M	99.87
	Our proposed model		0.35M	100
Lung and Colon Cancer	RCN	50	0.365M	99.68
	SEC		0.36M	99.79
	Reduced CNN		0.35M	99.89
	Our proposed model		0.35M	100

**Table 5:** Comparison of our proposed method results with other methods on the colon cancer detection dataset. “HI” and “CRAG” stand for histopathological images and colorectal adenocarcinoma gland, respectively.

Reference, Year	Models	Imaging	Dataset	Accuracy
[57], 2014	Neural Network	HI	Colonic Images	91.11
[58], 2014	CBIC	Biopsy Images	174 Biopsy Images	98.85
[59], 2014	DNN	HI	132 HI	96.30
[60], 2014	ANN	HI	21+28 HCC	90.2
[61], 2015	MLP, SMO, BLR	HT	Open Access	83.33
[62], 2015	SIFT, EFDs	Colon biopsy	Open Access	92.62
[63], 2015	CCD	Biopsy Images	Open Access	95.40
[64], 2015	Graph-SSL algorithm	HT	PPIs	80.7
[64], 2015	ANN, BNs, DTs	HT	PPIs	91.7
[65], 2016	DCNN	HI	Hematoxylin, HI	88, 100 (F1-score)
[66], 2016	CNNs	CT scans	56 patients	Sensitivity: 85
[67], 2016	Neural Network	CLE images	Endomicroscopies	Sensitivity: 85
[68], 2017	CNN, RF, kNN	CT scan	Open	87
[69], 2017	CNN autoencoders	HT	ETIS-LaribPolypDB	96.7
[70], 2017	CNNs	MRI-DWI	advanced rectal cancer	0.658, 0.99 (AUC)
[71], 2017	CNNs	BiopsyImages	Open Access	99.17
[72], 2018	RCCNet	HI	CRCHistoPhenotypes	80.61
[73], 2018	CNNs	HI	CRC samples	96
[74], 2018	Segnet	HI	Warwick-QU (A & B)	88.2 (A), 86.4 (B)
[75], 2018	SampEnMF	HI	Public Colorectal MRI	AUC: 0.983
[76], 2019	Random Forest	HI	Chang Gung, Taiwan	84, 0.82 (AUC)
[77], 2019	CNN	HI	NHI, Taiwan	Sensitivity: 0.837
[78], 2019	CNNs	Colonoscopy	Danish NSP	96.4, 97.1 (Sensitivity)
[14], 2019	CNN	Tissue slides	25 CRC patients	95
[71], 2017	CNNs	Biopsy Images	Open Access	99.17
[79], 2020	CNN	CT scans	10000-HI	99.6
[79], 2020	MFF-CNN	CT scans	NORM and TUM	96, 0.95 (F1-score)
[80], 2020	CNN	CT scans	CRAG	93.91
[81], 2020	CNN	CT scans	322 Images	94.8
[18], 2021	CNN + PCA	CT scans	LC25000	99.8
[18], 2021	ResNet, Inception	Slide Images	AiCOLO	96.98
[20], 2021	MobileNetV2	Colon cells	-	99.67
[82], 2021	IR-v2 Type 5	WSI	Chang Gung, Taiwan	F1-score, AUC: 0.99
[83], 2021	ResNet-18, VGG-19	Colonoscopy	-	98.3
[84], 2022	CNN	CT scans	Stoean and Kather	97.20
[21], 2022	CNN	CT scans	LC25000	99.50
[85], 2022	Deep Learning (DL)	CT scans	WSI	Sensitivity: 97.4
[86], 2022	ResNet	CT scans	TCIA	98.82, 98.28 (Sensitivity)
[87], 2022	CNN	CT scans	LC25000	100
[88], 2023	RNN, GoogLeNet	HI	Public Dataset	94.1, 97.5 (Sensitivity)
[89], 2023	ResNet	Colonoscopy	Public	99.8
[90], 2023	DL+AdaDelta	Tissue	Public Dataset	0.96
[91], 2023	ResNet50+Squeezenet	HI	Veterans’ Hospital	99.12, 99.34 (Sensitivity)
**Our, 2024**	**Our method**	CT scans	LC25000	Accuracy: **100**

**Table 6:** Comparison of our proposed result with other methods on the lung cancer detection dataset. “CAD”, “ML”, “GO”, and “LR” stand for clustering KNN-classifier, machine learning, genetic optimization, and logistic regression, respectively.

Reference, Year	Models	Imaging	Dataset	Accuracy
[92], 2013	SVM	CT scan	SUMS	Accuracy: 98.1
[93], 2014	SVM	CT scan	LIDC	Accuracy: 95.12
[94], 2014	CAD	CT scans	Radiological Data	Average: 98.9
[95], 2015	SVM	CT scan	Patients	Accuracy: 94.67%
[96], 2015	CAD	CT scan	LIDC	75.01, 83.35 (Sensitivity)
[97], 2015	Ensemble+ML	CT scan	LIDC	86.54
[98], 2015	CNNs	Chest X-rays	433 image dataset	AUC: 0.87–0.94
[99], 2016	DBN	CT scan	LIDC (174412 samples)	0.8119
[100], 2016	CADs and CNNs	CT scans	LIDC	Sensitivity: 78.9
[101], 2016	SVM+GO	CT scan	Medical imaging	Accuracy: 89.5
[102], 2016	Convolutional NN	CT scan	LIDC-IDRI	75.0
[103], 2016	Convolutional NN	CT scan	LIDC	Accuracy: 82.5
[104], 2017	ConvNet, SVM	CT scan	Danish DLCST trial	Accuracy: 72.9
[105], 2017	CNN, DNN, SAE	CT scans	LIDC-IDRI	84.15, 83.96 (Sensitivity)
[106], 2017	3D-CNNs	CT scan	Kaggle Data	Accuracy: 86.6
[107], 2017	CNN, DMN, SDAE	CT scan	LIDC	AUC: 0.899 ± 0.018
[108], 2017	Entropy Degradation	CT scan	NCI	Accuracy: 77.8
[109], 2018	VGG-network	CT scan	LIDC-IDRI	Accuracy: 95.60
[110], 2018	DenseNet-121	Chest X-rays	LIDC-IDRI	74.43, 74.68 (Sensitivity)
[111], 2018	Inception V3	CT scan	Genome Atlas	AUC: 0.733–0.856
[112], 2018	Otsu+ConvNet	CT scan	LIDC-IDRI	84.13, 91.69 (Sensitivity)
[113], 2019	Profuse clustering	CT scan	CIA	Accuracy: 98.42
[114], 2019	3D R-CNN	Chest X-rays	LIDC-IDRI	Sensitivity:94
[25], 2019	3D CNN	CT scan	Open-source image	Sensitivity: 84.4
[115], 2019	ODNN, LDA	CT scan	LIDC	94.56, 96.2 (Sensitivity)
[116], 2019	ANN	CT scan	Survey lung cancer	Accuracy: 96.67
[28], 2020	CNN	CT scans	LC25000	Accuracy: 97.20
[117], 2020	AlexNet, VGG19	LCDT images	I-ELCAP	96.25, 97.5 (Sensitivity)
[118], 2020	3D CNN	CT scans	LUNA16	Accuracy: 80
[119], 2020	AlexNet, VGG-16	CT scans	Open Dataset	Accuracy: 99.52
[18], 2021	Transfer learning	CT scans	LIDC	Accuracy: 99.12
[120], 2021	LCP-CNN	CT scans	US NLST	Sensitivity: 99
[121], 2021	AlexNet, GoogLeNet	CT scans	LIDC-IDRI	Precision: 100
[122], 2021	CNN	CT scans	Massachusetts Hospital	AUC: 0.71 (*p* = 0.018)
[123], 2021	Deep CNN, ReLU	Chest X-rays	Kaggle	Accuracy: 89.77
[35], 2022	MobileNetV2	CT scans	Public	Accuracy: 98.67
[124], 2022	SVM	CT scans	LIDC-IDRI	Accuracy: 94
[125], 2022	CNN-5CL	Chest X-rays	LIDC/IDRI	93.73, 98.88 (Sensitivity)
[126], 2023	2D-CNN	CT scans	LUNA16	Accuracy: 95
[37], 2023	LCP-CNN	Chest X-ray	Open	99.9, 99.89 (Specificity)
[45], 2023	LR+VGG16	CT scans	LC25000	99, 99 (Precision)
[46], 2023	EfficientNet-b4	CT scans	LC25000	Accuracy: 99.96
[47], 2023	GoogLeNet, VGG19	CT scans	LC25000	99.64, 99.85 (Sensitivity)
**Our, 2024**	**Our method**	CT scans	LC25000	Accuracy: **100**

**Table 7:** Comparison of our proposed result with other methods on lung and colon cancer detection dataset. Here, “IQ-OTHNCCD” is a lung cancer dataset.

Reference/Year	Models	Imaging	Dataset	Results
[127], 2020	CNN	CT scans	LC25000	Accuracy: 97.00
[41], 2020	InceptionV3, MobileNet	CT scans	LC25000	Accuracy: 99.91
[128], 2021	DHS-CapsNet	CT scans	LC25000	Accuracy: 99.23
[40], 2021	CNN, 2D Fourier	CT scans	LC25000	Accuracy: 96.33
[42], 2021	Capsule Network	CT scans	LC25000	Accuracy: 99.58
[129], 2021	DarkNet-19	CT scans	LC25000	Accuracy: 99.69
[44], 2022	AlexNet	CT scans	LC25000	Accuracy: 98.4
[130], 2022	DenseNet121, Random Forest	CT scans	LC25000	Accuracy: 98.6 F1-score: 0.985
[23], 2022	A Hybrid Ensemble Model	CT scans	LC25000	Accuracy: 99.3
[131], 2022	PCA + CNN + SVM, FHWT + CNN + SVM	CT scans	LC25000	Accuracy: 99.5 Accuracy: 99.6
[43], 2022	XGBoost	CT scans	LC25000	Accuracy: 99 F1-score: 98.8
[43], 2022	MobileNetV2, InceptionV2	CT scans	LC25000	Accuracy: 99.95
[39], 2023	Capsule Network	CT scans	LC25000	Accuracy: 99.32
[132], 2023	CNN	CT scans	LC25000	Accuracy: 99.76
[133], 2023	CNN	CT scans	LC25000	Accuracy: 98.96
[47], 2023	ANN	CT scans	LC25000	Sensitivity: 99.85 Precision: 100 Accuracy: 99.64
[45], 2023	Logistic Regression Model	CT scans	LC25000	Accuracy: 99.00 Precision: 99.00 Recall: 98.80 F1-score: 98.80
[134], 2024	SqueezeNet	CT scans	LC25000	Accuracy: 99.58
[135], 2024	EfficientNetB6 VGG19 InceptionResNetV2 DenseNet201 MobileNetV2	CT scans	LC25000	Accuracy: 93.12 Accuracy: 98.00 Accuracy: 97.92 Accuracy: 99.12 Accuracy: 99.32
[136], 2024	LightGBM	CT scans	LC25000	Accuracy: 100
**Our, 2024**	**Our method**	CT scans	LC25000	Accuracy: **100**

## Data Availability

Data is publicly available [55] and the details are attached in the manuscript.
